# Supplementary material for: Evolutionary Comparisons of Chelonid Alphaherpesvirus 5 (ChHV5) Genomes from Fibropapillomatosis-Afflicted Green (Chelonia mydas), Olive Ridley (Lepidochelys olivacea) and Kemp’s Ridley (Lepidochelys kempii) Sea Turtles
Source: Animals (Basel). 2021 Aug 25;11(9):2489. doi: 10.3390/ani11092489 (PMC8465875; doi:10.3390/ani11092489)

ChHV5 genes ranked by selection pressure, gene level results from this study versus Morrison et al. 2018 results

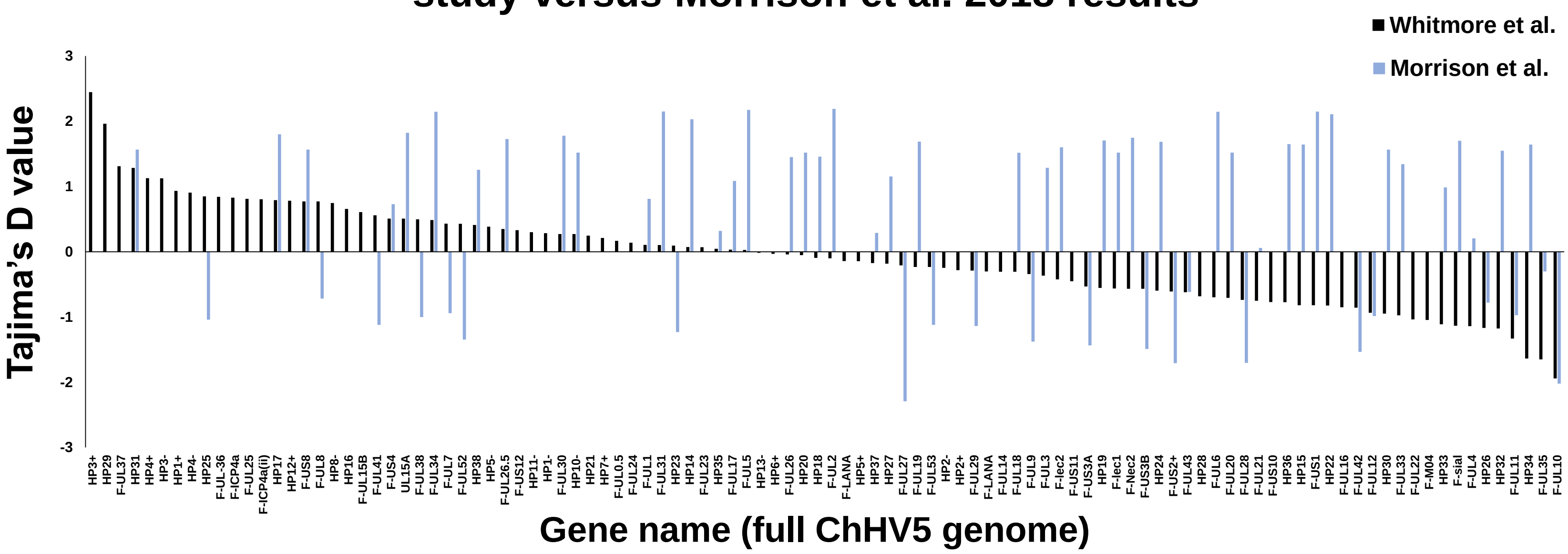

Supplement: Supplementary file 1 [file animals-11-02489-s001.zip › Whitmore et al Supplemental Files/Supplemental Figure 1.pdf]
